# Programa de Residência Médica em Cardiologia de Adultos do InCor em 2022: 40 Anos Formando Cardiologistas para as Demandas do Brasil

**DOI:** 10.36660/abc.20220457

**Published:** 2022-11-09

**Authors:** Marcos Pita Lottenberg, Luciana Dornfeld Bichuette, Luiz Aparecido Bortolotto, Luís Henrique Wolff Gowdak, Francisco Carlos da Costa Darrieux, Maria Angélica Binotto, Roberto Kalil, Bruno Caramelli

**Affiliations:** 1 Hospital das Clínicas Faculdade de Medicina Universidade de São Paulo São Paulo SP Brasil Instituto do Coração do Hospital das Clínicas da Faculdade de Medicina da Universidade de São Paulo , São Paulo , SP – Brasil

**Keywords:** Cardiologia, Residência Médica, Educação Médica

## Abstract

**Fundamento:**

Diante de dados demográficos referentes às áreas de atuação dos cardiologistas no Brasil, a coordenação do Programa de Residência Médica em Cardiologia do Instituto do Coração (PRM INCOR) entendeu a necessidade de uma atualização de seu conteúdo programático, a fim de adaptar o processo de formação à realidade profissional do cardiologista.

**Objetivo:**

O presente artigo tem como objetivo descrever à comunidade científica as atualizações recentemente implementadas no PRM INCOR.

**Métodos:**

No artigo, descrevemos as atualizações recentes do PRM INCOR, comparando a grade teórica pregressa e a atual. Expomos também o racional por trás de tais mudanças com dados de literatura relacionados à atuação do médico cardiologista no mercado de trabalho.

**Resultado:**

Houve uma redução da carga horária destinada a estágios de terapia intensiva, e um incremento nas atividades ambulatoriais relacionadas a medidas de prevenção primária e secundária. Além disso, o programa passou por uma reformulação de seu conteúdo didático, organizado agora por núcleos de competência.

**Conclusão:**

A atualização da grade curricular decorre da necessidade de adequar o PRM INCOR à realidade atual do mercado de trabalho brasileiro. O grupo envolvido na atualização está ciente que se trata de um processo dinâmico e que pode exigir modificações no decorrer do tempo.

## Introdução

As doenças cardiovasculares representam uma grande preocupação no Brasil e no mundo, sendo a principal causa de morte no país e responsável por uma parcela significativa dos custos relacionados aos cuidados de saúde. No entanto, muitos brasileiros ainda não recebem cuidados cardiovasculares adequados, seja por escassez de recursos ou formação deficitária de médicos especialistas. Melhorar a formação dos profissionais de saúde pode contribuir para mudar esse cenário, embora a compreensão das mudanças necessárias nos programas de treinamento em cardiologia não seja uma tarefa fácil.

Traçar paralelos entre os programas de residência em cardiologia no Brasil e em países desenvolvidos pode ajudar a identificar possíveis alvos de melhoria. Porém, é fundamental conhecer a inserção e a área de atuação dos profissionais de saúde depois de formados, para adaptar o processo de formação à realidade do mercado.

Segundo Scheffer et al., ^1^ em 2020, o número de médicos aproximou-se a meio milhão no Brasil, o que corresponde à relação de 2,4 por mil habitantes. Desses, 4,1% (n=17 802) são cardiologistas, o que coloca essa especialidade entre as 10 com o maior número de médicos, correspondendo à razão de 8,47 por 100.000 habitantes. Apesar do aumento do número de médicos, resultado da abertura de novos cursos de medicina, os profissionais ainda se encontram mal distribuídos geograficamente (entre regiões urbanas, periféricas e rurais) e no sistema de saúde (entre os setores público e privado, e entre os níveis de atenção primária, ambulatorial e hospitalar). ^1^ Ainda segundo este levantamento, apenas 8,16% dos cardiologistas em atividade possui o título de especialista em medicina intensiva. Apesar de não ser obrigatória a obtenção do título de especialista em terapia intensiva para realizar plantões em Unidades de Terapia Intensiva (UTI) e prontos-socorros no país, esse dado pode indicar que, o plantão nesses locais seja apenas uma etapa transitória na vida profissional do cardiologista no Brasil. Por outro lado, a preferência ou a maior duração dos estágios de emergência e UTI na grade curricular da residência, em detrimento de outras áreas clínicas, talvez não reflita o perfil profissional atual.

A Sociedade Brasileira de Cardiologia (SBC) realizou, em 2017, um levantamento entre seus sócios para identificar o perfil de atuação profissional do médico cardiologista no território brasileiro. ^2^ Um total de 2101 médicos responderam ao questionário, sendo que 70,5% eram portadores do título de cardiologia pela SBC e 29,5% eram aspirantes ao título; 49,3% declararam trabalhar em três ou mais locais diferentes, e 46,5% declararam ter o hospital público como local de trabalho mais frequente. Esse dado sugere que a formação do cardiologista, fundamentalmente em um hospital-escola público, traz ao médico residente aspectos práticos do cenário em que provavelmente será inserido após o término de sua formação. Outro estudo observacional transversal ^3^ envolvendo médicos graduados pela Faculdade de Medicina da Universidade de São Paulo (FMUSP) demonstrou que mais da metade deles trabalha tanto no setor público quanto privado, sendo que 63,4% trabalhavam em consultórios e clínicas particulares.

Este artigo tem como objetivo descrever as mudanças instituídas no programa de Residência Médica (PRM) do Instituto do Coração do Hospital das Clínicas da Faculdade de Medicina da Universidade de São Paulo (PRM Incor).

### Programa de residência médica

O PRM Incor existe desde 1982, quando aconteceu o credenciamento junto à Comissão de Residência Médica (COREME). Desde então, já foram formados 796 médicos cardiologistas vindos de todos os estados do Brasil. O PRM Incor tinha uma grade curricular que contemplava, no primeiro ano, dois meses de treinamento em urgência e emergência, um mês de treinamento em métodos diagnósticos e o restante do ano em estágios que envolviam atividades em ambulatório e enfermaria. O residente percorria as unidades de aterosclerose, coronariopatia crônica, valvopatias, insuficiência cardíaca, transplante cardíaco, hipertensão, lipídios e marcapasso. O segundo ano do programa também possuía estágios de cunho ambulatorial, como o de arritmia, miocardiopatias e doenças da aorta e cardiopatias congênitas. Além disso, havia grande concentração de atividades de terapia intensiva: cinco meses voltados exclusivamente a atividades na unidade coronariana, na UTI clínica e na UTI pós-operatória. A tabela 1 exemplifica a grade curricular em vigor até o ano de 2021.

**Tabela 1:** Grade curricular do programa de residência médica do Instituto do Coração em vigor até 2021

Primeiro Ano	Segundo Ano
Urgência e emergência (2 meses)	UTI clínica (2 meses)
Valvopatias (2 meses)	Unidade coronariana (2 meses)
Aterosclerose e coronariopatia crônica (2 meses)	UTI pós operatória (1 mês)
Estimulação cardíaca artificial (1 mês)	Miocardiopatias (1 mês)
Transplante cardíaco (1 mês)	Cardiopatias congênitas (1 mês)
Insuficiência cardíaca (1 mês)	Arritmia clínica (1 mês)
Métodos gráficos (1 mês)	Interconsulta (1 mês)
Hipertensão (15 dias)	Estágio eletivo (1 mês)
Lipídios (15 dias)	Doenças da Aorta (15 dias)
	Cardio-oncologia (15 dias)

*UTI: unidade de terapia intensiva.*

Apesar de se tratar de uma grade curricular extremamente competente e abrangente, a nova coordenação do programa decidiu realizar uma atualização considerando que é fundamental que o conteúdo programático da residência reflita a prática do médico cardiologista contemporâneo no país. Este processo envolveu longo período de discussão desenvolvido pelo grupo constituído por diretores de todas as unidades clínicas da instituição, incluindo docentes da Universidade de São Paulo que atuam na formação de médicos residentes há várias décadas e que estão indicados ao final deste artigo.

**Figura 1:**
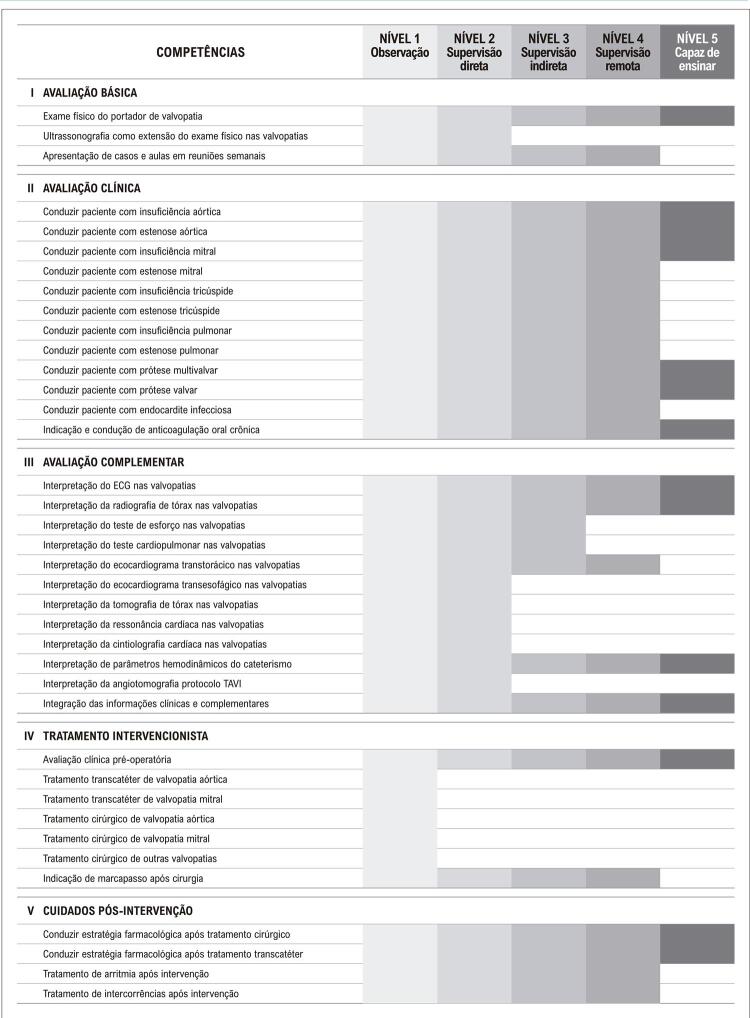
Competências pré-estabelecidas pelo núcleo de valvopatias. TAVI: implante de válvula aórtica transcateter.

**Figura 2:**
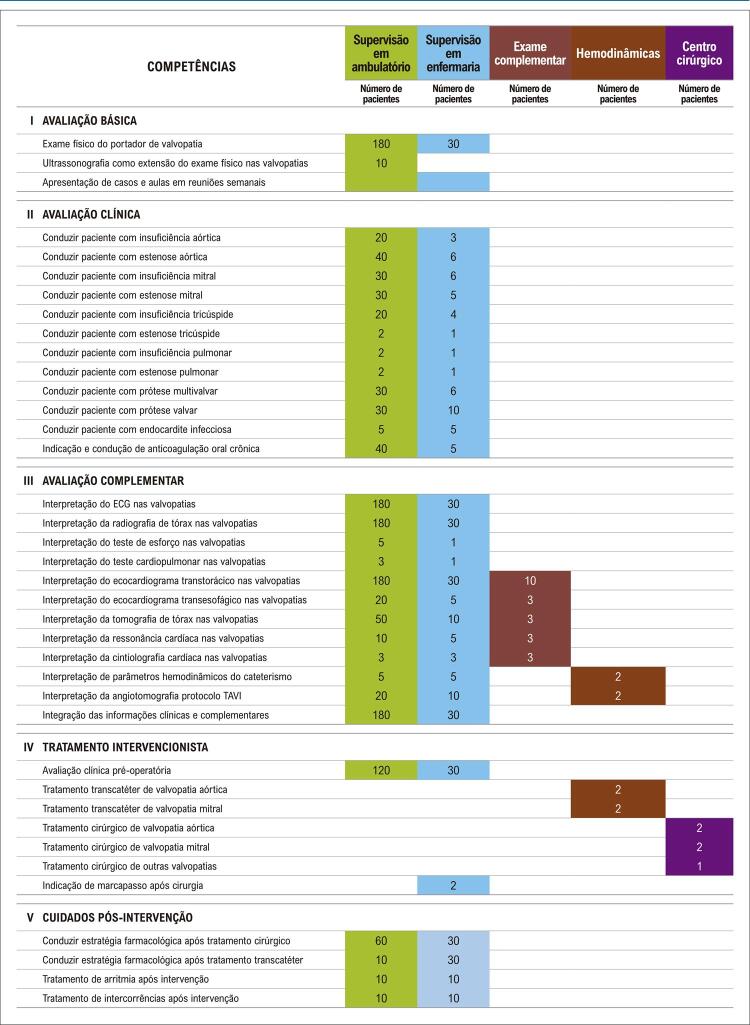
Estimativa de pacientes acompanhados no núcleo de valvopatias. TAVI: implante de válvula aórtica transcateter.

**Figura 3:**
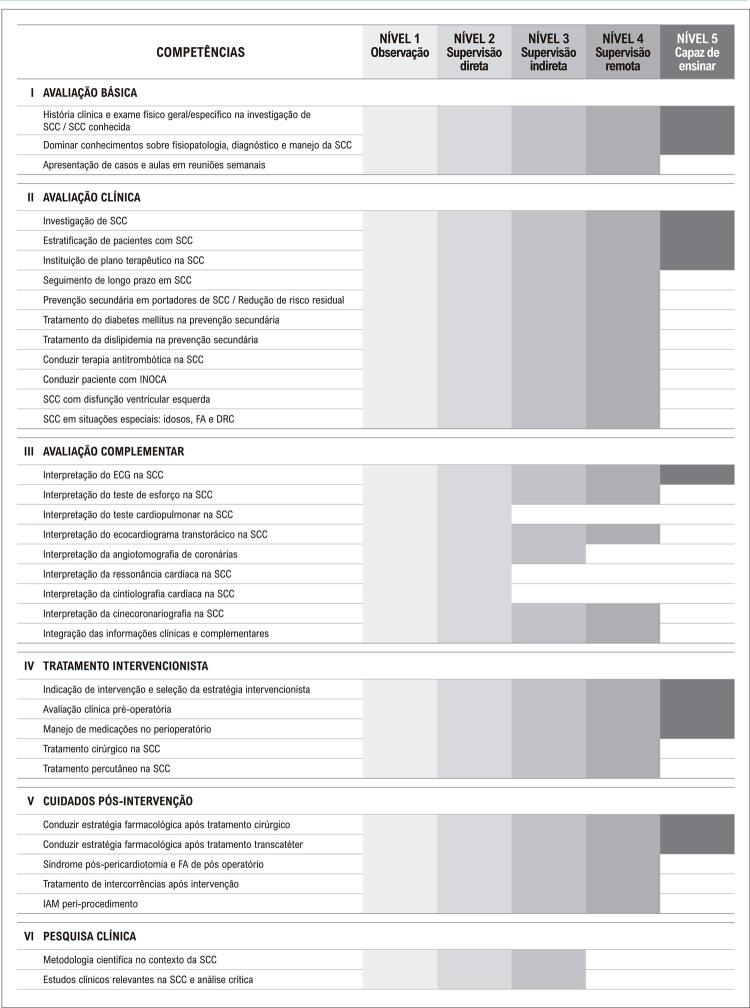
Competências pré-estabelecidas pelo núcleo de coronariopatia crônica/aterosclerose. INOCA: isquemia sem doença arterial coronariana; SCC: síndrome coronária crônica; FA: fibrilação atrial; IAM: infarto agudo do miocárdio; DRC: doença renal crônica.

Neste sentido, alguns pontos de mudança foram definidos: primeiramente, decidiu-se pelo desenvolvimento dos estágios em núcleos de competência, para proporcionar uma melhor organização e continuidade dos temas abordados. Essa dinâmica possibilita, também, que os núcleos unifiquem programas didáticos e organizem de forma mais centralizada as competências primordiais ao residente naqueles estágios. As Tabelas 2 e 3 e as Figuras 1 , 2 , 3 e 4 trazem o exemplo do conteúdo programático e das competências definidas pelos núcleos de valvopatias e coronariopatia crônica/aterosclerose.

**Tabela 2:** Programa teórico do núcleo de valvopatias

Número da Aula	Conteúdo da Aula
1	Diagnóstico e conduta nas valvopatias
2	Fisiopatologia das valvopatias
3	Febre reumática
4	Estenose aórtica
5	Insuficiência aórtica
6	Estenose mitral
7	Insuficiência mitral
8	Tratamento da valvopatia tricúspide
9	Doença coronariana e valvopatias
10	Endocardite infecciosa
11	TAVI
12	Fragilidade no portador de valvopatias
13	Amiloidose e estenose aórtica
14	Anticoagulação
15	Complicações da TAVI
16	Diretrizes
17	Revisão de 120 questões
18	Revisão geral

*TAVI: implante de válvula aórtica transcateter.*

**Tabela 3:** Programa teórico do núcleo de coronariopatia crônica/aterosclerose

Número da Aula	Conteúdo da Aula
1	Fisiopatologia da placa aterosclerótica
2	Interpretação da cinecoronariografia
3	Bases do tratamento medicamentoso
4	Redução de risco residual em diabéticos
5	Anticoagulação na SCC
6	Antiagregação em longo prazo na SCC
7	TAPD - seleção e duração
8	Etiologias incomuns de SCC
9	Indicações de revascularização
10	Seleção de estratégia de intervenção
11	SCC e disfunção ventricular
12	Investigação e estratificação da SCC
13	Síndrome coronária crônica em populações especiais
14	Uso de AngioTC de coronárias e RM cardíaca na SCC
15	Ecocardiograma de stress em SCC
16	Medicina Nuclear em SCC
17	Redução de risco residual lipídico
18	Reabilitação em SCC
19	Aspectos técnicos da cirurgia de revascularização
20	Escolha dos enxertos na cirurgia de revascularização
21	Aspectos técnicos da intervenção coronária percutânea
22	Tratamento da angina refratária

*SCC: síndrome coronária crônica; TAPD: terapia antiplaquetária dupla; AngioTC: angiotomografia.*

**Figura 4:**
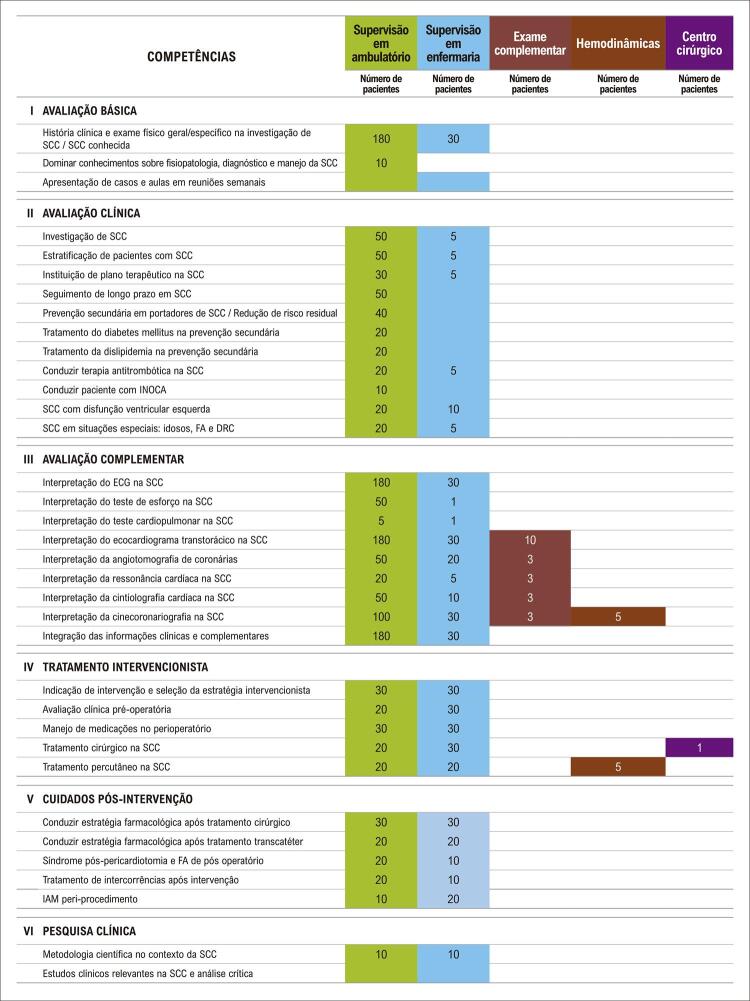
Estimativa de pacientes acompanhados no núcleo de coronariopatia crônica/aterosclerose. INOCA: isquemia sem doença arterial coronariana; SCC: síndrome coronária crônica; FA: fibrilação atrial; DRC: doença renal crônica.

**Figura 5:**
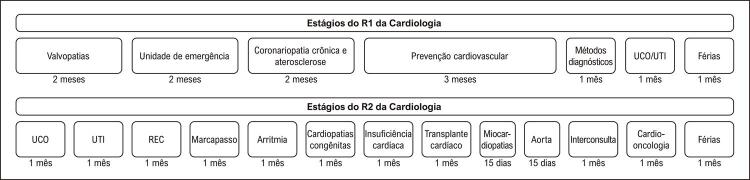
Grade curricular do programa de residência médica do Instituto do Coração a partir de 2022. UTI: Unidade de Terapia Intensiva; UCO: Unidade Coronariana; REC: UTI pós-operatória.

Em segundo lugar, entendeu-se que a carga horária destinada a estágios de terapia intensiva era desproporcional à carga de atuação do médico cardiologista nessa área, e optou-se pela redução do tempo de estágio em UTI. Em terceiro, diante da relevante participação do médico cardiologista em atividades ambulatoriais, e dado que essas contemplam em boa parte a assistência a pacientes em busca de medidas de prevenção primária e secundária, criou-se o núcleo de prevenção. Nesse, o residente tem a oportunidade de ter contato com áreas não contempladas no programa até então, como cardiogeriatria, reabilitação cardiopulmonar e ambulatório de triagem, além de estágios pré-existentes como lípides, hipertensão, tabagismo e ambulatório de atendimento a pacientes com cardiopatias em nível secundário em unidade externa. A Figura 5 mostra a nova grade curricular em vigor a partir de 2022.

### Formas de ingresso

Para se tornar cardiologista no Brasil, é necessário, após a formação em Medicina, cursar dois anos de especialização em Clínica Médica, seguidos de dois anos de especialização em Cardiologia. Até o ano de 2021 havia duas formas de ingresso no Incor. A primeira ocorria por meio do processo seletivo de Residência Médica, cuja prova é a mesma para todos as especialidades clínicas oferecidas pela instituição, com remuneração prevista durante o período e 28 vagas ofertadas. A segunda ocorria por meio de um processo seletivo exclusivo do Incor, cujos ingressantes possuíam uma menor carga horária de trabalho e algumas diferenças em relação à grade curricular, sem remuneração prevista. Esses profissionais não recebiam automaticamente, ao término de seu treinamento, o certificado de cardiologista pelo Conselho Federal de Medicina, sendo necessário realizar a prova de título da Sociedade Brasileira de Cardiologia.

Diante da necessidade de homogeneização na formação dos médicos na instituição, foi solicitado no ano de 2022, junto ao Ministério da Saúde e à Secretaria de Saúde do Estado de São Paulo, o aumento do número de vagas no PRM INCOR, que passou a ofertar 52 vagas, sendo que todos os aprovados passam atualmente por mesma grade curricular. Diante disso, foram extintas outras formas de ingresso.

## Conclusão

Entendemos que, para adequar o PRM em cardiologia do Incor à realidade do mercado de trabalho brasileiro, houve a necessidade de atualização da grade curricular, com destaque para atividades ambulatoriais de prevenção primária e secundária, que representam hoje parcela significativa de atuação do médico cardiologista no Brasil.

O grupo envolvido na gestão do PRM INCOR está ciente de que se trata de um processo dinâmico e que pode exigir modificações. O PRM INCOR já foi implementado e está em constante monitoramento por uma Comissão criada nos anos anteriores para acompanhar e resolver demandas de médicos residentes e professores do programa.
