# Supplementary material for: Programa de Residência Médica em Cardiologia de Adultos do InCor em 2022: 40 Anos Formando Cardiologistas para as Demandas do Brasil
Source: Arq Bras Cardiol. 2022 Nov 9;119(5 Suppl 1):43–51. [Article in Portuguese] doi: 10.36660/abc.20220457 (PMC9750193; doi:10.36660/abc.20220457)
Supplement: Translation

# Translation: Incor Residency Program in Adult Cardiology in 2022: 40 Years Preparing Cardiologists for the Demands in Brazil

**Authors:** Marcos Pita Lottenberg, Luciana Dornfeld Bichuette, Luiz Aparecido Bortolotto, Luís Henrique Wolff Gowdak, Francisco Carlos da Costa Darrieux, Maria Angélica Binotto, Roberto Kalil, Bruno Caramelli

**Affiliations:** 1 Brazil Instituto do Coração da Faculdade de Medicina da Universidade de São Paulo, São Paulo, SP – Brazil

**Keywords:** Cardiology, Medical residency, Medical Education

## Abstract

**Background:**

Considering demographic data related to the cardiologist’s fields of work in Brazil, the administrative board of the InCor medical residency program decided for an update of its curriculum content, to adapt the educational process to the cardiologist’s work reality.

**Objective:**

This article aimed to describe the recent updates applied to the InCor medical residency program.

**Methods:**

In the article, we described the recent updates on the InCor residency program, and compared the current curriculum track with the previous one. We also presented the rationale for these changes, based on the literature on the participation of cardiologists in the labor market.

**Results:**

There was a reduction in the working hours of residents in training in the intensive care unit, and an increase in the outpatient activities of primary and secondary prevention. Also, the didactic content was reformulated and became organized by the corresponding division.

**Conclusion:**

The update of the curriculum track of the InCor medical residency program was required in order to adapt it to the Brazilian labor market. The commission in charge of this update is aware that this is a dynamic process that may need changes over time.

## Introduction

Cardiovascular diseases are a serious concern in Brazil and in the world, representing the main cause of death in Brazil, and responsible for a high proportion of health costs. However, a large part of the Brazilian population still does not receive adequate cardiovascular care, due to scarcity of resources and poor training of specialist physicians. A better education of health professionals can help to change this scenario, although understanding the necessary changes in the cardiology fellowship programs is not an easy task.

Drawing a parallel between cardiology residency programs in Brazil and in developed countries can help identify potential improvement targets. However, it is essential to identify the physician’s placement and main field of work after graduation, to adapt the process of professional formation to the market reality.

According to Scheffer et al.,^1^ in 2020, there were nearly half million physicians in Brazil, corresponding to 2.4 doctors per thousand inhabitants. Of these, 4.1% (n=17,802) are cardiologists, placing Cardiology among the top ten specialties with the highest number of physicians (8.47 per 100,000 inhabitants). Despite the increasing number of professionals, resulting from the emerging of new undergraduate medical courses, the number of professionals is unequally distributed across geographical regions in Brazil (between urban, peripheral and rural areas) and across the health system (between the private and the public sectors, and between primary, outpatient and hospital care).^1^ According to the same study,^1^ only 8.16% of professionally active cardiologists have a title of intensive care specialist. Although working shifts in the emergency department and in intensive care units (ICUs) does not require a title of specialist, this may suggest that working shifts in these locations represent only a transitory stage of their professional lives in Brazil. On the other hand, the preference for and the longer duration of internship in emergency medicine, in detriment of other areas, in the cardiology residency program, may not reflect the current professional profile of cardiologists.

In 2017, the Brazilian Cardiology Society (SBC) conducted a survey among its members to identify the professional profile of cardiologists in Brazil.^2^ A total of 2101 physicians filled out the questionnaire; 70.5% had obtained the title of cardiology specialist from the SBC and 29.5% had applied for the title; 49.3% reported to work at three or more different places, and 46.5% reported that the public hospital was the most common place of work. This indicates that the cardiology training program is mostly carried out in public hospital schools, where the physician learns much of the practical aspects of the scenario in which he/she will be probably inserted after finishing the residency. Another cross-sectional observational study^3^ involving physicians who graduated from the University of Sao Paulo Medical School showed that more than half of them worked both in the private and the public sectors, and 63.4% worked in private offices and clinics.

This article aims to describe the changes implemented in the medical residency program (MRP) of the Heart Institute (InCor) of the University of Sao Paulo Medical School.

### Medical residency program

The Incor MRP was created in 1982, when it was accredited by the Residency Committee (COREME, *Comissão de Residência Médica* ). Since then, 796 physicians, coming from all Brazilian federative units, completed the cardiology fellowship program. The curricular track of the Incor MRP comprised, in the first year, of two months of training in urgency and emergency, one month of training in diagnostic methods, and nine months of training in outpatient and inpatient care. The intern passed through the units of atherosclerosis, chronic heart disease, valve disease, heart failure, heart transplant, hypertension, lipids and pacemaker. The second year of the program was comprised of an internship in outpatient care of patients with conditions like arrythmia, cardiomyopathies, aortic disease and congenital heart disease. In addition, there was a great number of activities focused on intensive care – five months exclusively dedicated to the training at the coronary care unit, at the medical ICU and the postoperative ICU. Table 1 exemplifies the curriculum in force until 2021.

**Table 1:** Curriculum of the University of Sao Paulo Medical School Institute of Heart (InCor) medical residency program in force until 2021

First year	Second year
Urgency and emergency (two months)	Medical ICU (two months)
Heart valve disease (two months)	Coronary care unit (two months)
Atherosclerosis and chronic coronary disease (two months)	Postoperative ICU (one month)
Artificial cardiac pacing (one month)	Myocardiopathies (one month)
Heart transplant (one month)	Congenital heart disease (one month)
Heart failure (one month)	Clinical arrhythmia (one month)
Graphical methods (one month)	Referral (one month)
Hypertension (15 days)	Elective internship (one month)
Lipids (15 days)	Aortic diseases (15 days)
	Cardio-oncology (15 days)

*ICU: intensive care unit.*

Although it is an extremely competent and comprehensive program, the new coordinators of the InCor MRP have decided for an update, considering that it is essential that the curricular content reflects the practice of the contemporary cardiologist in Brazil. This process involved a long period of discussion by the group composed of directors of the clinical units of the institution, including lecturers (listed at the end of this paper) of the University of Sao Paulo who have worked in residency programs for decades.

Then, some change points have been defined: first, it was decided that each stage of the residency would be developed at the respective unit/center to enable better organization and continuity of the topics. This dynamic process would also allow the unification of didactic programs across the centers and the concentration of the main competencies for the interns in each stage. Tables 2 and 3 and Figures 1-4 present an example of the program and competencies defined by the heart valve disease and chronic coronary disease/atherosclerosis centers.

**Table 2:** Didactic training content of the heart valve disease unit

Class number	Class content
1	Diagnosis and management of valve heart disease
2	Pathophysiology of valve heart disease
3	Rheumatic fever
4	Aortic stenosis
5	Aortic insufficiency
6	Mitral stenosis
7	Mitral insufficiency
8	Treatment of tricuspid valve disease
9	Coronary disease and heart valve disease
10	Infective endocarditis
11	TAVI
12	Frailty in patients with heart valve disease
13	Amyloidosis and aortic stenosis
14	Anticoagulation
15	Complications of TAVI
16	Guidelines
17	Review of 120 questions
18	General review

*TAVI: transcatheter aortic valve implantation.*

**Table 3:** Didactic training content defined by the unit of chronic coronary disease/atherosclerosis

Class number	Class content
1	Pathophysiology of the atherosclerotic plaque
2	Interpretation of coronary angiography
3	Fundamentals of pharmacological treatment
4	Reduction of residual risk in diabetics
5	Anticoagulation in CCS
6	Long term antiaggregation in CCS
7	DAPT – selection and duration
8	Uncommon causes of CCS
9	Indications for revascularization
10	Selection of the intervention strategy
11	Chronic coronary syndrome and ventricular dysfunction
12	Investigation and stratification of CCS
13	CCS in special populations
14	Use CCTA and CMR in CCS
15	Stress echocardiography in CCS
16	Nuclear medicine in CCS
17	Reduction of residual lipid risk
18	Rehabilitation in CCS
19	Technical aspects of revascularization surgery
20	Graft selection in revascularization surgery
21	Technical aspects of percutaneous coronary intervention
22	Treatment of refractory angina

*CCS: chronic coronary syndrome; DAPT: Dual antiplatelet therapy; CCTA: Coronary computed tomography angiography; CMR: cardiac magnetic resonance*

**Figure 1:** Competencies pre-established by the division of heart valve disease. TAVI: transcatheter aortic valve implantation.

**Figure 2:** Estimative of patients seen at the unit of heart valve diseases. TAVI: transcatheter aortic valve implantation.

**Figure 3:** Competencies pre-established by the division of chronic coronary disease/atherosclerosis. INOCA: ischemia and no obstructive coronary artery disease (INOCA); CCS: chronic coronary syndrome; AF: atrial fibrillation; AMI: acute myocardial infarction; CRD: chronic renal disease.

**Figure 4:** Estimative of patients seen at the unit of chronic coronary disease/atherosclerosis. INOCA: ischemia and no obstructive coronary artery disease; CCS: chronic coronary syndrome; AF: atrial fibrillation; CRD: chronic renal disease.

Second, the total workload in intensive care units determined in the fellowship program was considered disproportionate given the actual working participation of cardiologists in this area, and the total of hours the residents spent in the ICU were then reduced. Third, in light of the active participation of cardiologists in outpatient care and considering that it involves strategies of primary and secondary prevention, the division of prevention was created. In this unit, the intern has the opportunity to gain work experience in areas that were not covered by the program so far, such as geriatric cardiology, cardiopulmonary rehabilitation and outpatient screening, in addition to pre-existing fellowship, including lipids, hypertension, smoking, outpatient care of heart disease patients at secondary care level at an external clinic. Figure 5 presents the new curricular program that entered into force in 2022.

**Figure 5:** Curricular program of the Incor residency program implemented in 2022. ICU: intensive care unit; CCU: coronary care unit.

### Entry pathways

In Brazil, to become a cardiologist, after graduating from medical school, the physician must complete two years of internal medicine residency, followed by two years of cardiology fellowship. Until 2021 there were two entry pathways in the InCor residency program. The first one was a selection process consisting of an exam that is the same for every clinical specialty of the institution, with a salary predicted for the entire residency period and 28 openings. The second one consisted of a selection process that was exclusive of the Incor. The interns had a lower workload, there were some differences in the rotations and no salary predicted. These physicians who have fully completed their training do not get a certificate in cardiology from the Brazilian National Medical Council, and still have to pass the Brazilian Cardiology Society exam.

Considering the need to homogenize medical residency training at InCor, in 2022, the number of openings increased to 52, as requested and conceded by the Brazilian Ministry of Health and Sao Paulo State’s Secretariat of Health. All residents, once passed the exam, follow the same curricular program, and hence other entry pathways were excluded.

## Conclusion

To adapt the residency program in cardiology offered at InCor to the labor market in Brazil, we believed that an update of the curricular program was required, focusing on outpatient activities of primary and secondary prevention, which accounts for a considerable volume of the cardiologist practice in the country.

The administrative board of the InCor MPR understands that this is a dynamic process that may need modification. The InCor MRP was already implemented and has been constantly monitored by a commission created in previous years to follow-up and meet the demands of resident physicians and teachers of the program.
